# LOMA: A fast method to generate efficient tagged-random primers despite amplification bias of random PCR on pathogens

**DOI:** 10.1186/1471-2105-9-368

**Published:** 2008-09-10

**Authors:** Wah Heng Lee, Christopher W Wong, Wan Yee Leong, Lance D Miller, Wing Kin Sung

**Affiliations:** 1 Genome Institute of Singapore, 60 Biopolis Street #02-01, Genome, Singapore; 2 Department of Computer Science, National University of Singapore, 10 Kent Ridge Crescent, Singapore

## Abstract

**Background:**

Pathogen detection using DNA microarrays has the potential to become a fast and comprehensive diagnostics tool. However, since pathogen detection chips currently utilize random primers rather than specific primers for the RT-PCR step, bias inherent in random PCR amplification becomes a serious problem that causes large inaccuracies in hybridization signals.

**Results:**

In this paper, we study how the efficiency of random PCR amplification affects hybridization signals. We describe a model that predicts the amplification efficiency of a given random primer on a target viral genome. The prediction allows us to filter false-negative probes of the genome that lie in regions of poor random PCR amplification and improves the accuracy of pathogen detection. Subsequently, we propose LOMA, an algorithm to generate random primers that have good amplification efficiency. Wet-lab validation showed that the generated random primers improve the amplification efficiency significantly.

**Conclusion:**

The blind use of a random primer with attached universal tag (random-tagged primer) in a PCR reaction on a pathogen sample may not lead to a successful amplification. Thus, the design of random-tagged primers is an important consideration when performing PCR.

## Background

Pathogen detection has become an important part of research in diagnostics and drug discovery. To this day, the accurate and sensitive detection of infectious disease agents is still thwarted with difficulties. Detection tools are typically designed from sequence information stored in public databases. However, as some viruses mutate or recombine, their sequence information may become inaccurate. Moreover, sequence information for novel pathogens such as severe acute respiratory syndrome (SARS) will not be available until much later. Detection tools will most probably fail when such scenarios happen.

Traditionally, pathogen detection is performed using techniques such as in vitro cultures, immunologic assays and PCR. However, these approaches are time-consuming, labor-intensive and can only detect a limited number of pathogens at one time. Furthermore, a clinical prediction of the infectious source would have to be made before any of the above diagnostic techniques can be conducted [1]. Clearly, a fast and accurate detection and identification of the infectious etiologic agents responsible for disease would lead to new or earlier treatments and even prevention strategies.

In recent years, oligonucleotide microarrays have been used to detect, identify and even discover viral pathogens [2-4]. A pathogen detection microarray usually contains 20–70 mer DNA fragments (known as probes) designed from a pre-determined set of viral pathogens. Every probe is chosen such that it is thermodynamically optimal, does not form secondary structures and hybridizes only to its target viral genome [5,6]. In a typical experiment to detect the presence of viral pathogens in a given sample, the sample first undergoes reverse-transcription polymerase chain reaction (RT-PCR) using tagged random primers [7]. Then, the amplified cDNA is end labeled and hybridized onto the microarray. Ideally, probes specific to the viral pathogens present in the given sample would have significantly higher signal intensity than that of all other probes.

In practice, despite efforts to design "good" probes that avoid cross-hybridizations, have optimal melting temperatures and contain no secondary structures [8], "bad" probes (i.e. probes that should hybridize with sample but do not, and probes that should not hybridize but do) are still prevalent. Several explanations have been suggested for the occurrence of these phenomena. For example, DNA hybridization may be sequence-dependent causing some probes to be inherently noisy [9]. Another likely explanation is that probes designed for a particular virus may no longer hybridize due to the highly mutagenic nature of the virus [10].

One possible explanation, that is often overlooked, is the failure of random-primed RT-PCR amplification to amplify the target regions of the probes [11,12]. So far, previous research has focused on improving specific primers [13]. In this paper, we provide insights into how random primers work and its implications for hybridization signals. We build on our previous paper [14] describing in further detail the AES algorithm that identifies genomics sequences that can be successfully amplified by random primers, facilitating the design of appropriate microarray probes for detection of the pathogen. Finally, we introduce LOMA, a novel algorithm for designing efficient tagged-random primers. Note that the PCR technology described in this paper is specifically suited for RNA virus detection as the detection of DNA viruses would not require the RT-PCR step.

### Tagged Random Primer Amplification

In diagnostic laboratories, specific detection of a small number of pathogens is performed using pathogen-specific primers in separate RT-PCR assays. While microarrays allow for the parallel detection of hundreds of pathogens in one assay, there are significant technical and bioinformatics challenges for RT-PCR using specific primers.

Therefore random primers have traditionally been used for the unbiased amplification of samples in microarrays [2,7,15]. A tagged random primer consists of two parts: a constant 17 bp at the 5'-end known as the 5' tag and a random oligomer (unknown base N) of length 9–15 at the 3'-end which could theoretically bind to any sequence [16]. In a RT-PCR assay, the first (reverse transcription) step uses the tagged random primer to generate 500–1000 bp products with tagged primer sequences at both ends. In the second (PCR) step, the primer with the constant 17 bp sequence is used to amplify the PCR products from the first step.

### Genome-wide Amplification Bias

**Figure 1:**
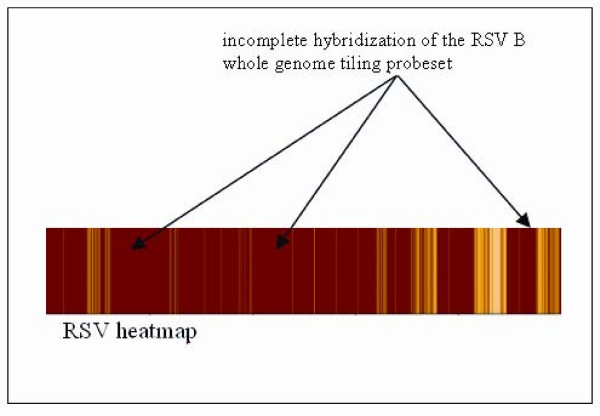
**Heatmap of probe signal intensity for a RSV B sample following random RT-PCR**. Red regions correspond to probes that did not have signal intensities above threshold. As probe signal intensity increases, the heatmap changes from red to orange to yellow to white.

Recently, we have published a pathogen detection approach using microarrays based on random primer amplification [14]. In our paper, we reported an observation that experiments using random priming amplification often resulted in incomplete hybridization of the pathogen genome marked by interspersed genomic regions not detected by tiling probes on the microarray (Figure 1.). In fact, this phenomenon has also been reported by several others [17,18].

Further analysis on the probes and RSV B genome rule out sequence polymorphisms, probe GC content and genome secondary structure as significant causes of this phenomenon. This suggests that a PCR-based amplification bias due to differential binding of the random primers to different parts of the viral genome at the reverse transcription (RT) step could be the main cause of incomplete hybridization. This differential binding behavior of random primers could be influenced by the presence of intra-primer secondary structure formation (ie the 5'-end tag forms a dimer or hairpin with the 3'-end random oligomer) or melting temperatures [19].

One way to avoid hybridization inaccuracies is to refrain from designing probes or filter probes from potential regions of a viral genome where amplification by a given random primer is likely to fail. We develop a model of the random primer amplification process on a viral genome to predict the regions where amplification may fail. Using this model, we compute an Amplification Efficiency Score (AES) for each position of the given genome to predict its binding affinity with the given tagged random primer. Regions of the given genome with high AES are predicted to have good binding affinity with the given tagged random primer and thus would be ideal locations to design probes from. Conversely, regions of the given genome with low AES are predicted to have poor binding affinity with the given tagged random primer. We should avoid designing probes from such regions.

Although we have the capability to predict the binding affinities of a given tagged random primer to a given genome, it would be most useful if we know the tagged random primer that binds to the given genome optimally. However, computing the AES for all possible tagged random primers with the given genome to obtain the optimal primer is impractical.

In the following sections, we describe experiments that provide further evidence that intra-primer secondary structure formation resulting in differential binding of the random primers is indeed the main reason behind incomplete hybridization. Subsequently, we propose a fast and practical algorithm to generate a tagged random primer that is able to optimally amplify a set of given genomes.

## Results

### Generating the Tag of a Random Primer using LOMA

Amplification failure may occur if there are many regions of the target genome where the tagged random primer cannot bind. As such, using any available commercial random primer or a random primer that was used in other publications may not guarantee a successful amplification on a target genome. In the previous section, we have introduced the AES and described how it allows us to compare the amplification efficiency of different random primers on a target genome.

The best way to obtain the most efficient tagged random primer to amplify a target genome is to compute the AES graph for all possible combinations of the 17-bp 5'end tag and choose the tag that has the highest average AES with the target genome. This is impractical as this would require 4^17 ^runs of the AES computation algorithm. A naïve approach would be to randomly generate a large number of tags (eg. 10000) and choose the one that has the highest average AES with the target genome. Other similar randomization approaches could also be used to improve the chances of getting a more efficient tag to amplify the target genome. However, these approaches are still slow especially when we need to choose an efficient random-tag primer for multiple genomes.

We propose LOMA (Least Occurrence Merging Algorithm), a more deterministic and faster algorithm to generate an efficient tag for a target genome *v*_*a*_. The idea is to use a "divide and conquer" strategy to generate *n*-bp tags by concatenating *m *shorter *k*-mers where *m *= *n*/*k*. Recall that the 5'end tag of the random primer should be not similar to *v*_*a *_to avoid mispriming. To form such a tag, the consistuent *k*-mers should also be dissimilar to *v*_*a*_. Based on this criterion, we compute the number of occurrences with more than 75% similarity in *v*_*a *_for each of the 4^*k *^*k*-mers. Then, we sort the *k*-mers based on their occurrence count in *v*_*a *_in ascending order. Tags are generated using the top ranking *k*-mers whose number of occurrences in *v*_*a *_is lower than some threshold *T*. Ideally, we want to generate tags using only *k*-mers with no occurrence in *v*_*a*_, ie *T *= 0.

**Figure 2:**
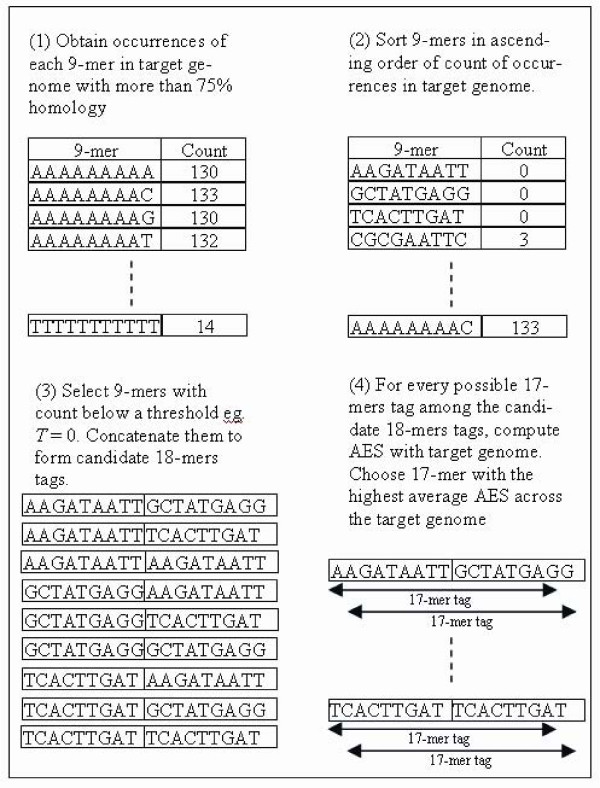
**Flowchart of LOMA**. Flowchart of LOMA with n = 17, k = 9 and T = 0.

Suppose *x k*-mers have occurrences in *v*_*a *_less than *T*. We generate a tag by concatenating any *m *of the *x k*-mers. This results in *x*^*m *^possible tags. Since *x *is small and *m *is typically 2 or 3, the total number of tags generated is much less compared to a brute force or randomized approach. Furthermore, the *x*^*m *^tags generated by our method are guaranteed to be dissimilar to *v*_*a*_. Thus, all there is left to do is to compute the AES with *v*_*a *_of each of the *x*^*m *^tags and choose the one with the highest average AES across *v*_*a*_. Figure 2. shows the flowchart of our algorithm.

Unlike randomized approaches, LOMA is easily extended to generate an efficient tag for multiple genomes. Specifically, given a set of genomes V, we need only to modify step one of the algorithm to compute the number of occurrences with more than 75% similarity in every genome in V for each of the 4k k-mers. Once candidate random-tags are generated, we compute their AES with each of the genomes in V and choose the one with the highest average AES for all the genomes in V.

### Experimental Results

We describe experiments to test the hypothesis that different tagged-random primers have different amplification efficiencies and to assess the effectiveness of our algorithm to generate a good tagged-random primer. In our experiments, we use eight human nasopharyngeal aspirate patient samples obtained from childen under 4 years of age with lower respiratory tract infections. Using real-time PCR with specific primers, we confirmed that five samples contain human respiratory syncytial virus (RSV) while the remaining three samples contain human metapneumovirus (HMPV) [14].

Three tagged-random primers are then used to amplify the eight samples:

1. Primer A1 (5'-GTT TCC CAG TCA CGA TAN NNN NNN NN-3'): A commercially available tagged-random primer used in many publications. [20-22]

2. Primer A2 (5'-GAT GAG GGA AGA TGG GGN NNN NNN NN-3'): Primer with highest AES among 10000 randomly generated tags.

3. Primer A3 (5'-TAG GTC GGT CGG TAG GTN NNN NNN NN-3'): Primer generated using our proposed algorithm LOMA.

Subsequently, the samples are hybridized onto our pathogen detection chip. Since our pathogen detection chip contains tiling 40-mer probes of both RSV and HMPV, the number and distribution of the probes with high signal intensities would give a good indication of the amount of PCR products generated across the target genome by a tagged-random primer. We expect that a tagged-random primer with desirable amplification efficiency that generates sufficient PCR products uniformly across the whole target genome would result in high signal intensity probes distributed evenly across the whole genome.

We present the first set of experiments involving the amplification of five RSV patient samples by the three random-tagged primers A1, A2 and A3 [see Additional file 1]. In each experiment involving a particular pair of RSV patient sample and random-tagged primer, hybridization signal intensities for the 1948 probes tiled across the 15225 bp RSV genome were compared to their corresponding AES along the genome. When using primer A1, we obtained AES with values less than 5000 with an average of 3300. However, when primers A2 and A3 are used, the AES averages are 110000 and 140000, respectively. This dramatic increase in predicted amplification efficiency gave an indication that in theory, our designed random-tagged primers A2 and particularly A3 perform much better than A1.

**Figure 3:**
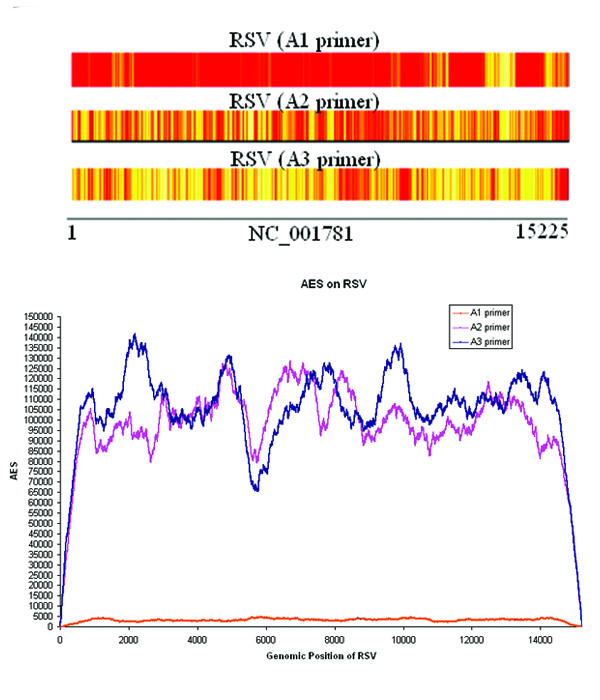
**Application of AES on a RSV sample**. An RSV patient sample was amplified separately using primer A1, primer A2 and primer A3. Hybridization signals of probes after amplification by each primer are shown as a heatmap. The probes that have detectable signals above threshold are shown in orange/yellow in the corresponding heatmaps. The graph below the heatmaps shows our AES prediction for the three primers: A1 (orange line), primer A2 (pink line) and primer A3 (dark blue line). Our AES predictions closely matches the actual hybridization results, ie primer A3 performs slightly better than primer A2 but both A3 and A2 performs significantly better than A1 on RSV.

Recall that probes in regions of high AES are expected to be least affected by a poor amplification and thus have the correct high hybridization signals if the pathogen is present in the sample. For all the experiments, we observed that high AES significantly correlates to probe hybridization signal intensity above the detection threshold with a p-value of 2.2 × 10^-16 ^using the Fisher's exact test. About 80% of the probes with high signal intensities (≥ mean + 3 standarad deviation) have high AES values. We also observed that primers A2 and A3 showed a tremendous improvement in overall PCR efficiency in amplifying RSV over primer A1. This increase in PCR efficiency resulted in increased hybridization of DNA to the probes and is reflected in the uniformly higher signal intensities observed using primer A2 and A3. This is illustrated in Figure 3. Further analysis of the RSV experiments revealed that only 20% to 30% of the 1948 RSV probes had signal intensities above detection threshold when primer A1 was used. By contrast, the use of primer A2 resulted in 60% to 71% of probes with signal intensities above detection threshold. Primer A3 fared slightly better than primer A2, resulting in more than 70% of the probes having signal intensities above detection threshold.

**Figure 4:**
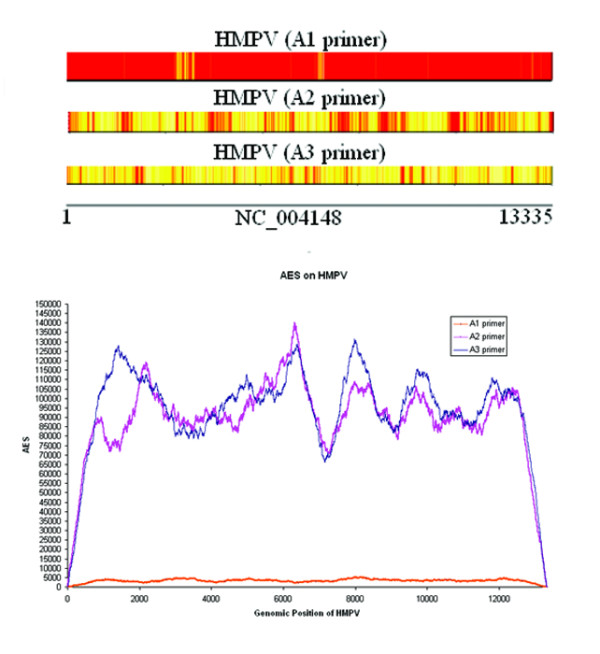
**Application of AES on a HMPV sample**. An HMPV patient sample was amplified separately using primer A1, primer A2 and primer A3. Hybridization signals of probes after amplification by each primer are shown as a heatmap. The probes that have detectable signals above threshold are shown in orange/yellow in the corresponding heatmaps. The graph below the heatmaps shows our AES prediction for the three primers: A1 (orange line), primer A2 (pink line) and primer A3 (dark blue line). Our AES predictions closely matches the actual hybridization results, ie primer A3 performs slightly better than primer A2 but both A3 and A2 performs significantly better than A1 on HMPV.

We conducted another set of experiments to verify that the observations made involving RSV and the three random-tagged primers are not isolated observations and that they can be replicated in other genomes as well. Following the experimental procedure used in the previous set of experiments, three patient samples containing HMPV are subjected to amplification by primers A1, A2 and A3 [see Additional file 2]. Similarly, in each experiment involving a particular pair of HMPV patient sample and random-tagged primer, hybridization signal intensities for the 1705 probes tiled across the 13335 bp HMPV genome were compared to their corresponding AES along the genome. Figure 4. shows the heatmaps and AES plots of the HMPV genome when amplified by primers A1, A2 and A3. The results are similar to that of the first set of experiments on RSV. In the three samples, Primer A1 performs worse on HMPV than RSV, causing only < 8% of the 1705 probes to be detected above threshold. Primers A2 and A3 performed much better than primer A1, causing >80% and > 88% of the probes to be detected above threshold respectively.

Our experiments have shown that the commonly used primer A1 amplify RSV and HMPV poorly. Further analysis reveals that many instances of the primer A1 that are supposed to bind to RSV and HMPV form self-dimers and hence unable to amplify the genome efficiently. On the other hand, primers A2 and A3 amplified RSV and HMPV efficiently. However, compared to primer A2, primer A3 was generated in a much shorter time by LOMA and performs just as well, if not better.

## Discussion

### Multiplexing Random-Tagged Primers

LOMA generates random-tagged primers that are capable of amplifying their target genomes efficiently with coverage of more than 70% up to 90%. We explore the possibility of using multiple random-tagged primers to achieve a more complete amplification of the target genome.

**Figure 5:**
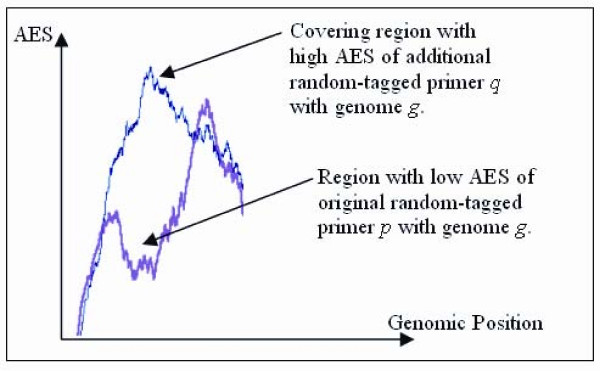
**Design of multiple random-tagged primers to amplify a target genome g**. Original primer p has a region with low AES on g. We design additional primer q such that it has high AES in that region.

In our experiments, we observed that one random-tagged primer may amplify a particular region of a target genome more efficiently than another random-tagged primer. For example in Figure 4. at genomic positions 1500–1900 of HMPV, the heatmap shows that primer A3 performs much better than primer A2. On the other hand, on the same genome at positions 2000–2200, the heatmap shows that primer A2 performs better than primer A3. This suggests that it is possible to design multiple collaborating random-tagged primers to amplify a target genome. The idea is to design additional random-tagged primers that have regions with high AES covering the regions with low AES of existing random-tagged primers. This is shown in Figure 5.

Although this approach is highly viable as suggested by our experimental results, achieving a successful multiplexing of multiple random-tagged primers in the laboratory may not be as straight-forward as the traditional multiplexing of specific primers [23]. Recall that a random-tagged primer consists of random oligomers that could theoretically bind to all possible sequences. Using two or more random-tagged primers simultaneously in a PCR amplification reaction may result in the formation of primer-dimers among all instances of the random-tagged primers and cause the amplification to fail. To ensure higher success of multiplexing random-tagged primers, an alternative solution is to perform the PCR reaction with the first random-tagged primer, then perform another PCR reaction with the second random-tagged primer and so on. In other words, we multiplex *n *random-tagged primers by performing *n *PCR reactions in series. This will avoid the problem of primer-dimers when multiplexing random-tagged primers.

## Conclusion

New generation pathogen detection chips need to be able to detect a wide range of known pathogens and potentially novel pathogens. As it is not cost-effective to design specific primers for all the pathogens on the chip and quite impossible to design specific primers for yet to be known pathogens, random primer amplification is preferred over primer-specific amplification. However, genome-wide amplification bias of random primers is a serious yet commonly overlooked problem.

In this paper, we built on our previous paper and described a model to predict the amplification efficiency of a random-tagged primer given a target genome(s). The AES provided us with a measurement that we can use to compare the amplification efficiency of different random-tagged primers on the target genome. This paved the way for the development of LOMA, a fast and effective random-tagged primer generator. Through experiments, we have shown that the random-tagged primer generated by LOMA performs significantly better than a commonly used random-tagged primer on different genomes. Furthermore, LOMA is able to generate good tagged-random primers much faster than randomized approaches.

Unlike specific primers that are almost always selected from the target genome under stringent primer design criteria [24], people tend to use random primers without checking their suitability with the target genome. This is a serious oversight that may cause inaccuracies in downstream work such as microarray analysis. Our research has shown that the blind use of a random-tagged primer in a PCR reaction on a pathogen sample may not lead to a successful amplification. Thus, the design of random-tagged primers is an important consideration when performing PCR and should be a common practice when using random-tagged primers.

LOMA is implemented in java and is available at 

## Methods

### Microarray Design

Using complete genome sequences of 35 human viruses downloaded from the NCBI Taxonomy Database [25], we generated 40-mer probes tiled across each genome and overlapping at an average 8-base resolution. Seven replicates of each probe were synthesized at random positions on the microarray using Nimblegen proprietary technology [26]. In addition, 10000 random probes with 40–60% GC-content were added to the microarray to assess background signal levels for quality control. Additional controls included 400 probes to human immune genes (positive controls) and 162 probes of a plant virus, PMMV (negative control). In total, 390482 probes were hybridized onto the array.

### Sample Preparation and Hybridization

Nasopharyngeal washes were obtained from an Indonesian pediatric population using a standardized WHO protocol as described [27]. The patients, aged between 0–48 months, showed symptoms of lower respiratory tract infrections and were diagnosed with bronchiolitis or pneumonia when they visited the clinic between Feb 1999 till Feb 2001. The samples were stored at -80°C in RNAzol (Leedo Medical Laboratories, Inc., Friendswood, TX). RNA was later extracted from samples with RNAzol according to the manufacturer's instructions [28,29], resuspended in RNA storage solution (Ambion, Inc., Austin, TX) and frozen at -80°C until further use. RNA was reverse transcribed to cDNA using tagged random primers as described [7,30]. The cDNA was then amplified by random PCR, fragmented, end-labeled with biotin, hybridized onto the microarray and stained as previously described [31] with one exception: 0.82 M TMAC to Nimblegen's hybridization buffer to minimize nonspecific hybridization. Refer to our previous paper [14] for a detailed protocol description.

### Spot Intensity Analysis

Microarrays were scanned at 5 μm resolution using an Axon 400b scanner and Genepix 4 software (Molecular Devices, Sunnyvale, CA). Signal intensities were extracted using Nimblescan 2.1 software (NimbleGen Systems, Madison, WI). From the seven replicates of each probe, we computed the median and standard deviation signal intensity of the probe. The probe median signal intensities are then visualized through heatmaps and used to analyze the accuracy of our AES predictions.

### Amplification Efficiency Model of the RT-PCR Process

How well a primer pair binds to the target genome impacts RT-PCR efficiency. In the case of using random primers, the quality of the RT-PCR product depends on how well a random primer instantiation pair binds to the target genome. Here, we termed a particular configuration of the given random primer as a random primer instance. For example, GTTTCCCAGTCACGATA*TTTTAAAAG *and GTTTCCCAGTCACGATA*CATCATCAT *are instantiations of the random primer GTTTCCCAGTCACGATA*NNNNNNNNN*. Some instantiations of the random primer can bind better to the target genome than others. The identification of such random primer instantiations and where they bind to the target genome gives us an indication of how likely a particular region of the target genome will be amplified. Using this approach, we proposed an amplification efficiency model in our previous paper [14] which computes an Amplification Efficiency Score (AES) for every position of a target genome. We provide a more detailed description of our model here.

**Figure 6:**
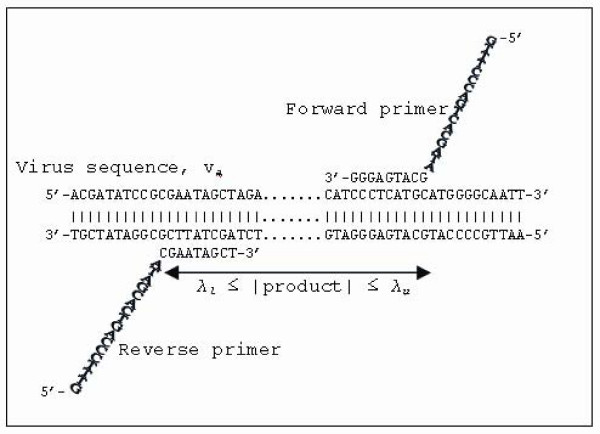
**RT-PCR binding process**. RT-PCR binding process of a pair of random primers on a target virus sequence v_a_.

As a concrete example for our modeling, we use a random primer that has a fixed 17-mer header and a variable 9-mer tail of the form (5'-GTT TCC CAG TCA CGA TAN NNN NNN NN-3'). This random primer is commonly used in virus detection experiments [20-22]. Let v_*a *_be the actual virus in the sample. To get a RT-PCR product in a region between positions *i *and *j *of v_*a*_, we require (1) a forward primer binding to position *i*, (2) a reverse primer binding to position *j *and (3) *λ*_*l *_≤ |*i *- *j*| ≤ *λ*_*u *_where *λ*_*l *_and *λ*_*u *_are the lower and upper bounds of the desired PCR product length respectively. Figure 6. shows a typical binding of a pair of random primers on a virus genome sequence.

Consider a pair of forward and reverse random primer instantiations where the forward primer is at a particular position *i *of *v*_*a*_, the reverse primer is at position *j *of *v*_*a *_and |*i *- *j*| is the product length. Let *P*^*f*^(*i*) and *P*^*r*^(*j*) be the probability that the forward primer can bind to position *i *and the probability that the reverse primer can bind to position *j *respectively. For simplicity, we assume that a random (forward or reverse) primer instantiation can bind to a particular position *i *of *v*_*a *_only if 9-mer of the instantiated random oligomers of the random primer is a reverse complement of the length-9 substring at position *i *of *v*_*a *_(Note that other binding criteria such as 75% similarity rule or nearest-neighbour binding free energy [5] can be used as well). Thus, all other instantiations of the given random primer whose instantiated random oligomers is not a subsequence of *v*_*a*_, do not contribute to the amplification process and thus omitted from the computation of AES. We compute *P*^*f*^(*i*) and *P*^*r*^(*j*) based on well-established primer design criteria [32]. The idea is that *P*^*f*^(*i*) and *P*^*r*^(*j*) will be small if the forward primer or reverse primer forms self-dimers or has extreme melting temperatures, or form a significant primer-dimer with each other. Another consideration is that if the fixed 17 basepairs 5-end tag of the given random primer is similar to *v*_*a*_, it may lead to mispriming and thus results in a lower *P*^*f*^(*i*) and *P*^*r*^(*j*) for all *i *and *j*.

It is difficult to assess the exact extent of influence of primer-dimers and melting temperatures on amplification. Hence, we estimate *P*^*f*^(*i*) and *P*^*r*^(*j*) using a simple model:

1. A primer cannot bind to the sequence efficiently if it folds onto itself. A primer is a self-dimer if it forms a 3' end or internal hairpin with three or more bases. Thus, *P*^*f*^(*i*) = 0 if the forward primer at *i *forms a self-dimer. Similarly, *P*^*r*^(*j*) = 0 if the reverse primer at *j *forms a self-dimer.

2. The RT-PCR process is performed at a certain temperature, normally 55°C–60°C. If the melting temperature of a primer is not at this ideal temperature, then the primer may not bind to the sequence. Hence, we model this observation by decreasing *P*^*f*^(*i*) and *P*^*r*^(*j*) proportionally to the difference in the melting temperature of the forward primer and reverse primer to the ideal experimental temperature respectively. Specifically, *P*^*f*^(*i*) = 1 - (|Tm(forward primer) - *TM*|/*TM*) and *P*^*r*^(*j*) = 1 - (|Tm(reverse primer) - *TM*|/*TM*) where *TM *is the ideal experimental temperature and Tm(*x*) is the melting temperature of a primer *x*. We compute Tm(*x*) of the primer *x *using the formula given in [33].

3. To avoid mispriming, if the 17 basepairs fixed tag of the random primer has more than 75% similarity to any subsequence of the target genome, we discard this random primer. That is, *P*^*f*^(*i*) = 0 if the forward primer at *i *has a fix tag with more than 75% similarity to any subsequence of the target genome. Similarly, *P*^*r*^(*j*) = 0 if the reverse primer at *j *has a fix tag with more than 75% similarity to any subsequence of the target genome.

Based on our model, the probability that a pair of random primer instantiations give a good quality PCR product from position *i *to *j *on *v*_*a *_is *P*^*f*^(*i*) × *P*^*r*^(*j*). Due to the abundance of random primer instantiations used in a RT-PCR process, it is likely that all pairs of random primer instantiations that can effectively bind to *v*_*a *_will contribute a PCR product. Thus, for a valid forward primer at position *i*, we must compute the above probabilities for a range of positions *j *at which a valid reverse primer exists, ie *λ*_*l *_|*i *- *j*| ≤ *λ*_*u*_. Thus, an Amplification Efficiency Score, *AES*_*x*_, for every position *x *of *v*_*a *_can be computed by considering the combined effect of all forward and reverse primer-pairs that amplifies it:

(1) $$AES_{x}=\sum_{i=x-\lambda_{u}}^{x}\left\{P^{f}(i)\times \sum_{j=\max(x+1,i+\lambda_{l})}^{i+\lambda_{u}}P^{r}(j)\right\}$$

Once we compute the AES for all positions of *v*_*a*_, we plot the AES against the genomic positions of *v*_*a*_. This generates a graph which indicates the regions in *v*_*a *_predicted to be amplified efficiently by the given random primer (represented by peaks) and regions that do not (represented by troughs). These regions in *v*_*a *_predicted to be amplified efficiently will be very useful in designing probes in a pathogen-detection microarray. Conversely, we should omit probes from regions in *v*_*a *_which are predicted not to amplify efficiently since we cannot tell if these probes did not hybridize due to the absence of *v*_*a *_in the sample or just that the amplification by the random primers failed.

Our model allows us to predict how successful the amplification on a target viral genome will be given a particular tagged random primer. An ideal tagged random primer would generate high AES values uniformly across the whole target genome. This quantification of the efficiency of amplification of a tagged random primer on a target genome in the form of AES also enables us to compare the effectiveness of different tagged random primers if they are to be used to amplify the genome. For example, random primer *r*_1 _is predicted to work better than random primer *r*_2 _if the average AES of *r*_1 _across a target genome is higher than that of *r*_2_. This implies that we can now design a tagged random primer that maximizes the amplification efficiency on a given set of target genomes.

## Authors' contributions

CWW and WYL carried out the amplification and microarray experiments for the study. WHL designed the algorithm, performed the analysis and drafted the manuscript. LDM and WKS conceived of the study, and participated in its design and coordination and helped to draft the manuscript. All authors read and approved the final manuscript.

## Supplementary Material

Additional file 1 The probe signal intensities of a RSV sample using 3 different random-tagged primers for RT-PCR. Click here for file

Additional file 2 The probe signal intensities of a HMPV sample using 3 different random-tagged primers for RT-PCR. Click here for file
